# The Role of the Human Hypothalamus in Food Intake Networks: An MRI Perspective

**DOI:** 10.3389/fnut.2021.760914

**Published:** 2022-01-03

**Authors:** Coleen Roger, Adèle Lasbleiz, Maxime Guye, Anne Dutour, Bénédicte Gaborit, Jean-Philippe Ranjeva

**Affiliations:** ^1^Centre de Résonance Magnétique Biologique et Médicale (CRMBM), Centre National de la Recherche Scientifique (CNRS), Université Aix-Marseille, Marseille, France; ^2^Centre d'Exploration Métabolique par Résonance Magnétique (CEMEREM), Assistance Publique-Hôpitaux de Marseille (AP-HM), Hôpital Universitaire de la Timone, Marseille, France; ^3^Département d'Endocrinologie, Assistance Publique-Hôpitaux de Marseille (AP-HM), Hôpital de la Conception, Marseille, France

**Keywords:** MRI, networks, anorexia, obesity, resting state, diffusion, hypothalamus

## Abstract

Hypothalamus (HT), this small structure often perceived through the prism of neuroimaging as morphologically and functionally homogeneous, plays a key role in the primitive act of feeding. The current paper aims at reviewing the contribution of magnetic resonance imaging (MRI) in the study of the role of the HT in food intake regulation. It focuses on the different MRI techniques that have been used to describe structurally and functionally the Human HT. The latest advances in HT parcellation as well as perspectives in this field are presented. The value of MRI in the study of eating disorders such as anorexia nervosa (AN) and obesity are also highlighted.

## Introduction

Energy expenditure appears as a continuous process, while energy refilling through food intake is by nature discontinuous. In order to keep the body fat mass stable, a mechanism guaranteeing an efficient balance between energy expenditure and energy intake is therefore needed (1). Modern ways of life in developed countries tend to disturb such energetic balance. Indeed, this phenomenon is leading to drastic increases in the prevalence of eating disorders such as obesity, often referred as “globesity” (2) or AN, which holds the highest mortality rate of any mental illness (3). Although it is true that eating behavior is influenced by our lifestyle, it is the result of the complex association of genetic, metabolic and neurological alterations that have not been completely elucidated to date.

**Figure 1:**
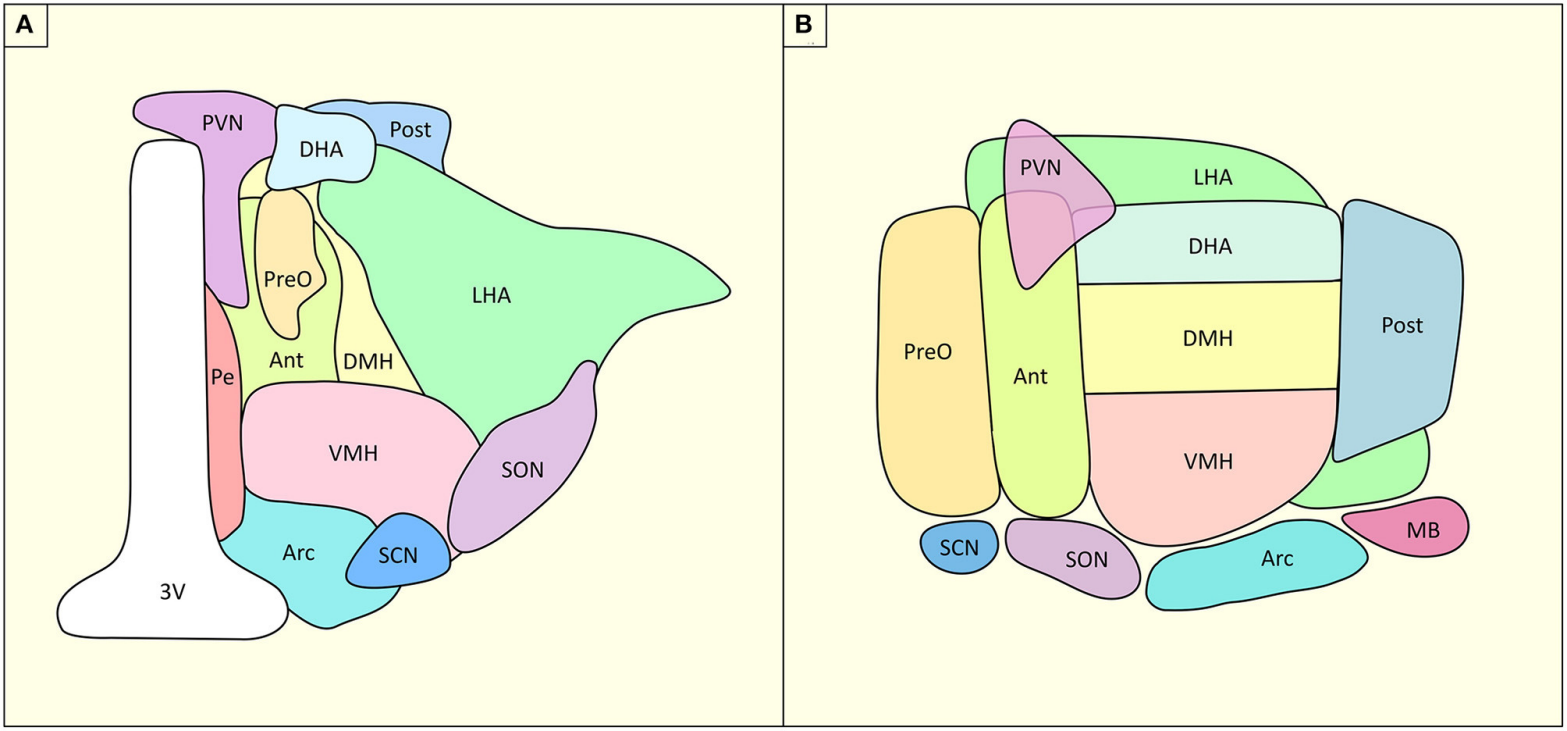
Structural organization of HT and its nuclei. **(A)** Coronal slice of the hypothalamus, **(B)** Sagittal slice of the hypothalamus. Ant, anterior hypothalamus; Arc, arcuate nucleus, DHA, dorsal hypothalamic area; DMH, dorsomedial hypothalamus; LHA, lateral hypothalamic area; MB, mammillary body; Pe, periventricular nucleus; Post, posterior hypothalamic nucleus; PreO, preoptic nucleus; PVN, paraventricular nucleus; SCN, suprachiasmatic nucleus; SON, supraoptic nucleus; VMH, ventromedial hypothalamus; 3V, third ventricle. Based on (4, 5).

One key structure in the control of the energetic balance is the hypothalamus (HT), a small and complex deep brain structure. HT is composed of four sub-regions: the pre-optic, anterior, tuberal and mammillary regions. These regions project to the autonomous system, and the primary and associative systems. They each host multiple nuclei which bear different physiological-anatomical characteristics. Along the rostral-caudal axis, HT is composed by, (i) at the anterior level: the preoptic nuclei (lateral and medial), the anterior hypothalamic nuclei, the paraventricular nuclei (PVN), the anteroventral periventricular nuclei, the supraoptic nuclei and the suprachiasmatic nuclei; (ii) at the medial level: the dorsomedial nuclei, the ventromedial nuclei, and the arcuate nuclei; (iii) laterally: the lateral nucleus and (iv) and at the posterior level: the posterior nucleus and the mammillary bodies (Figure 1). Each of these nuclei has specific roles in homeostatic regulation of energy, food intake, thirst but also temperature, sexual dimorphism, sleep and circadian rhythm (6).

**Figure 2:**
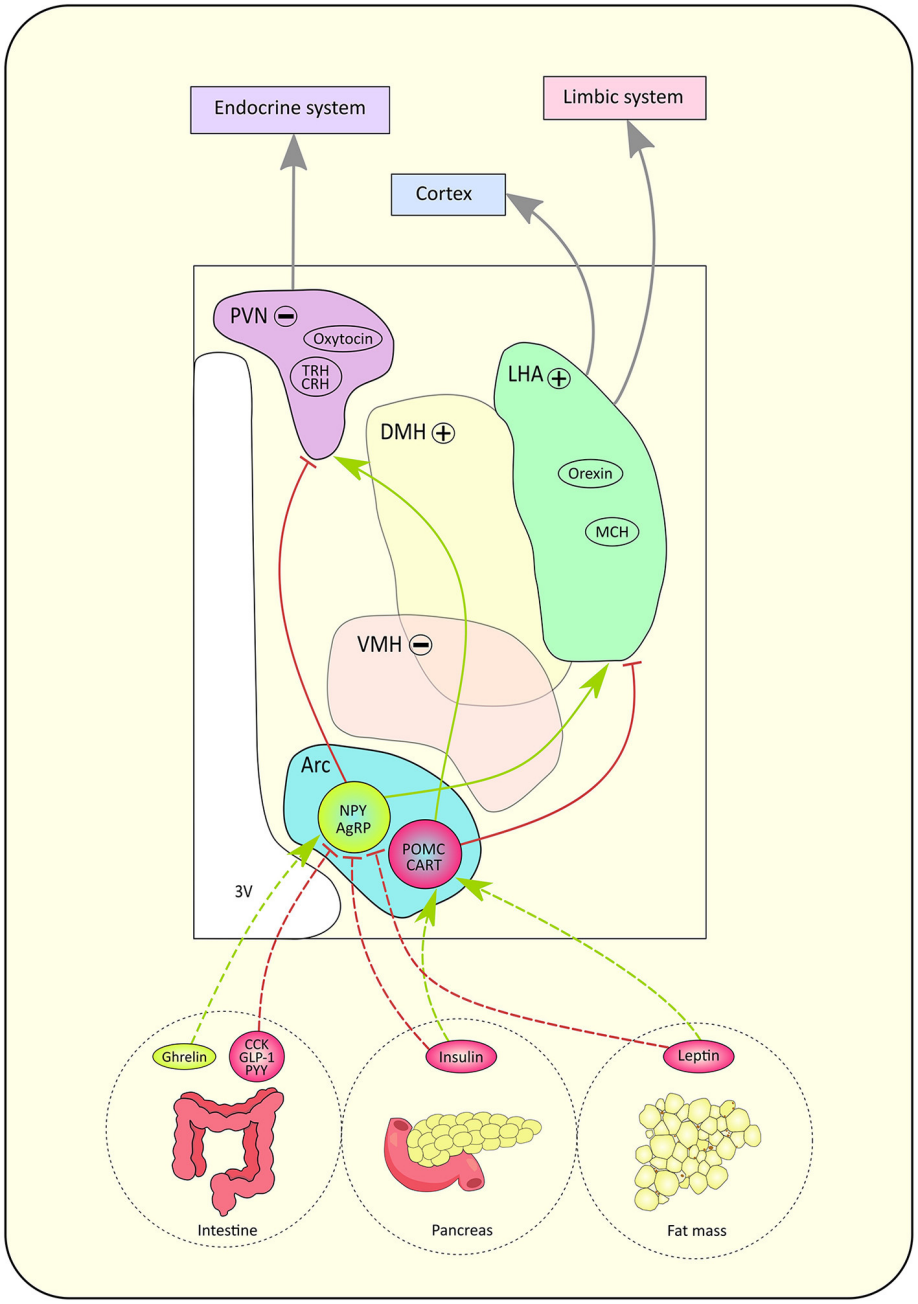
Schematic pathway of the lipostat and its peptidergic circuitry. According to hunger state, hormones are released by the intestine, pancreas and fat mass. Those signals directly influence neuronal activity in the hypothalamus through their action on antagonistic neurons located in the arcuate nucleus (Arc): orexinergic neurons expressing neuropeptide Y (NPY)/ agouti-related peptide (AgRP) and anorexinergic neurons expressing pro-opio-melanocortine (POMC)/ cocaine- and amphetamine- regulated transcript (CART). Gut hormones such as cholecystokinin (CCK), peptide YY (PYY), glucagon-like peptide 1 (GLP-1) inhibit NPY/AgRP neurons while ghrelin stimulates NPY/AgRP neurons. The pancreas secretes insulin while leptin is secreted by fat mass cells. Both hormones trigger the inhibition of NPY/AgRP neurons but the stimulation of POMC/CART neurons. Red arrows represent inhibitory connections, green arrows represent stimulatory connections, gray connections represent indirect pathways. The paraventricular nucleus (PVN) hosts neurons expressing oxytocin, thyrotropin-releasing hormone (TRH) and corticotropin-releasing hormone (CRH), while the lateral hypothalamic area (LHA) contains neurons expressing orexin and melanin-concentrating hormone (MCH). The '+' sign means the nucleus has an orexinergic effect on food intake when activated, whereas the '−' sign indicates that the nucleus has an anorexinergic effect on food intake when activated. Indeed, when activated, the dorsomedial hypothalamus (DMH) and lateral hypothalamic area (LHA) have an orexinergic action while the ventromedial hypothalamus (VMH) and paraventricular nucleus (PVN) have an anorexinergic action on food intake. Finally, the PVN sends information to the endocrine system while the LHA carries input to the cortex and limbic systems (24). 3V, third ventricle.

Most of the knowledge regarding the functional role of HT nuclei in food intake processes have been derived from animal studies (7–9). Figure 2 is an attempt to summarize the current knowledge on such networks. Pioneer works in rats showed that bilateral lesions of the ventromedial HT (VMH) induced hyperphagia while bilateral lesions of the lateral hypothalamic area (LHA) provoked hypophagia (11). These early lesion approaches led to the definition of VMH as the “center of satiety”, responsible for the food intake inhibition, and LHA as the “center of hunger”, whose role is to stimulate food intake (11). The PVN sends information to the endocrine system while the LHA carries input to the cortex and limbic systems (12).

Advances in deciphering such mechanisms were also possible through the characterization of a network called the ‘hypothalamic lipostat', which refers to the neuronal pathways involved in the integration of information related to food intake (13). HT ensures energy balance in the long term by integrating factors circulating by humoral or vagal pathways such as satiety signals and adipokines [see (10) for review]. More recently, the “gut-brain axis” was also associated to the regulation of food intake. Indeed, serotonin-secreting cells found in the gut communicate with the brain through the vagus nerve (14, 15). Visceral signals from the gut activate neurons in the nucleus of the solitary tract (NTS), which stimulates regions known to mediate feeding behavior, such as the lateral parabrachial nucleus (PBN) localized in the pons (16). The gut-brain axis is also involved in food reward (17) and sugar preference (18).

Five hypothalamic nuclei have been associated to food intake regulation: the lateral, ventromedial, dorsomedial, PVN and arcuate nuclei (19). The arcuate nuclei hosts first order neurons, which impose antagonistic effects on food intake. On one hand, neurons expressing the neuropeptide Y (NPY) and neuropeptide agouti-gene related peptide (AgRP) project to second order neurons localized in the PVN to stimulate appetite (12). On the other hand, neurons expressing proopiomelanocortin (POMC) and the neuropeptide cocain and amphetamine related transcript (CART) project to second order neurons contained in the LHA in order to inhibit food intake (12), although POMC neurons' role seems more contrasted than previously described [see (20) for review]. Depending on energetic state, different types of signals activate or inhibit these neurons. Indeed, when lipid reserves are high, plasmatic leptin concentration increases (21). Leptin is an anorexigenic hormone, which acts on food intake in the long term. It reduces appetite by increasing the activity of POMC/CART neurons and inhibiting NPY/ AgRP neurons (22). The regulation of food intake is ensured in the short and medium term by insulin as well. After a meal, insulin secretion rises and exercises an anorexigenic effect (22). Intestinal peptides also modulate appetite. For example, cholecystokinin (CCK), PYY 3–36 and glucagon-like peptide-1 (GLP-1) stimulate satiety whereas ghrelin promotes food intake (22). New hypothalamic regions have recently been shown to be involved in food intake mechanisms. For example, the tuberal lateral nucleus seems to promote an orexigenic effect on food intake by inhibiting the PVN through somatostatin (SST) interneuron signaling (23).

In addition, food intake is subjected to a non-homeostatic control influenced by the palatability of food as well as the environment and emotional state (24). Second order hypothalamic neurons constitute intermediate relays to some regions of the limbic system such as the amygdala, the hippocampus, the insula, the striatum and the orbitofrontal cortex (OFC). These structures, already known to be involved in emotions, memory or addictions, seem to have an important role in modulating eating behavior (22). Furthermore, the limbic system undergoes an inhibitory control from the prefrontal cortex which promotes meal termination (25).

HT dysfunction has long been implicated in eating disorders such as obesity (26) and anorexia nervosa (AN). With 650 million people affected worldwide in 2016, obesity is defined by a body mass index (BMI) over 30, resulting from an abnormal fat accumulation, which presents a risk to health (WHO). Rat studies showed that obesity induced by high fat diet led to a chronic low-grade HT inflammation with insulin and leptin resistance which promoted weight gain (27, 28). Besides, obese individuals showed T_2_ hyperintensity in the mediobasal HT, reflecting gliosis (29). More generally, HT appears to be an open window for some saturated fatty acids (SFAs) such as palmitate passing through the partially incomplete blood brain barrier in the arcuate nuclei and the median eminence (30–32). Furthermore, it was shown that the presence of SFAs in the HT induces neuronal responses leading to stimulated inflammatory cytokine expression, reactive oxygen species (ROS) production, endoplasmic reticulum stress and microglia recruitment [see (33) for review]. These findings collectively support the hypothalamic inflammatory hypothesis. Malfunction of the reward system has also been associated to a decreased availability of dopamine D2 receptor (D2R) proportionally to the BMI of patients (34).

Concurrently, AN affects approximately 4% of females and 0.3% males during their lifetime (35). This pathology is characterized by an important self-inflicted restriction of food intake leading to a BMI below 17.5, intense fear of gaining weight, as well as altered body perception influencing self-esteem and causing denial of the severity of current thinness (Diagnostic and Statistical Manual of Mental Disorders 5th Ed. (DSM-5) criteria http://www.dsm5.org/meetus/pages/eatingdisorders.aspx). In comparison to obesity, the cellular mechanisms and physiological substrates of AN have not been as thoroughly characterized (36). However, the administration of D2/3 receptors (D2/3R) antagonists to a rodent model of AN, the “activity-based anorexia” (ABA) model, was found to reduce weight loss and hypophagia, and increase survival (37). Furthermore, D2/3R density or affinity seem to increase in AN patients (38). As it is the case for obesity, this result support the existence of a dysfunction of the dopaminergic system in AN. Therefore D2/3R appear to be interesting therapeutic targets for AN. Furthermore, serotonin levels have been associated to almost all the behavioral changes observed in AN patients, such as extreme dieting weight loss, hyperactivity, depression/anxiety, self-control, and behavioral impulsivity (39). However, one of the most interesting hypothesis on the pathophysiology of AN is that chronic food restriction alters the ghrelin signaling pathway and leads to the development of abnormal behaviors such as addiction to food starvation (40).

Overall, translation of the cellular mechanisms observed in animal models to Human is not easy and straightforward. In that context, neuroimaging has appeared as a game changer to explore food intake and the consequences of its deregulation from an anatomical, functional or even metabolic point of view (41, 42). However, in order to define the precise function of each hypothalamic nucleus in human, it is necessary to clarify their location and their respective roles within the different functional brain networks. Given the small size of the HT, its location and the large number of hypothalamic nuclei, fine characterization of HT is still ongoing. This paper gives an overview of the different neuroimaging studies which have attempted to characterize the HT, as well as the different ways to progress in the field. We will also highlight the undeniable value of MRI in the study of eating disorders such as AN and obesity.

## Anatomical MRI Characterization, Parcellation and Morphometry of HT

HT is generally poorly defined or individualized in the numerous subcortical brain atlases proposed in the literature (43–48). Despite the fact that brain MRI stands out as the most suitable tool in the study of the central nervous system, the poor contrasts between hypothalamic nuclei has led to consider the HT as a single homogeneous structure, which prevents the precise morphological characterization of each nuclei. Moreover, hypothalamic MRI is subject to artifacts as a consequence of the vicinity of the HT with sinuses and bones (41). Nevertheless, standardized guidelines have been proposed for manual HT delineation as a single structure (49). Recently, a few MRI studies aimed at characterizing hypothalamic sub-regions based on anatomical references. The first neuroimaging atlas of the HT was based on 3D-MRI acquired at 1.5T in twenty healthy subjects (50). Thus, by combining anatomical, histological and magnetic resonance images, the human HT was parcellated into PVN, VMH, arcuate nuclei, and the posterior hypothalamic area (50).

One year later, combination of *in vivo* 1.5T MRI and *ex vivo* 7T MRI data allowed Makris and co-workers to individualize five hypothalamic sub-regions: the lower anterior HT, the upper anterior HT, the lower tuberical HT, the upper tuberical HT and the posterior HT (51). This approach was sufficiently robust to perform quantitative morphometry of HT sub-parts, showing an overall larger HT in men relative to women after correction for brain size. Indeed, significant differences in sizes were reported bilaterally in the five HT subregions, especially in the tuberal region which includes the LHA, infundibular, PVN, VMH, and supraoptic nucleus (51).

A more detailed parcellation was proposed by Lemaire and collaborators, based on a 1.5T MRI database (52). Using precise anatomical landmarks, especially the white matter (WM) bundles neighboring the HT, most internal hypothalamic structures were located: posterior nucleus, dorsomedial nucleus, infundibular nucleus, lateral nucleus, mammillary body, PVN, preoptic nuclei, suprachiasmatic nucleus, supraoptic nucleus, tuberomamillaris nucleus and VMH (52).

More recently, the first digital human brain atlas to gather neuroimaging, high-resolution histology, and chemo-architecture was created (53). This atlas has the particularity to be based on 7T MRI and 3T diffusion-weighted imaging (DWI) scans, as well as 1,356 large-format cellular resolution (1 μm/pixel) Nissl and immunohistochemistry anatomical plates from a complete adult female brain. Using this strategy enabled the description of every hypothalamic nuclei, including the most challenging structures to describe such as the suprachiasmatic nucleus.

However, a step forward was reached recently by Neudorfer and colleagues (4) who proposed a template based on a large set of 3T data composed by 990 3D-MPRAGE T_1_-weighted MRI (isotropic voxel size 1 mm^3^) of healthy controls from the Human Connectome Project database (https://www.humanconnectome.org/study/hcp-young-adult/document/1200-subjects-data-release). Using state-of-the-art multiscale template-building methods including non-linear registration procedures [ANTS, http://stnava.github.io/ANTs (54)], the authors proposed the first complete high-resolution *in-vivo* anatomical atlas of the human HT with a spatial resolution up to 0.25 × 0.25 × 0.25 mm voxel size. Based on this high resolved template, three experts were able to delineate the thirteen hypothalamic nuclei and individualized them. Projection to individual subjects using non-linear registration showed differences in volumes of hypothalamic nuclei which are dependent on brain hemisphere and subject gender in healthy subjects. These improvements in delineation and quantification were highly dependent on the artificially increased spatial resolution brought by the use of a large number of exams, which limited partial volume effects and allowed for a better contrast between HT nuclei.

**Figure 3:**
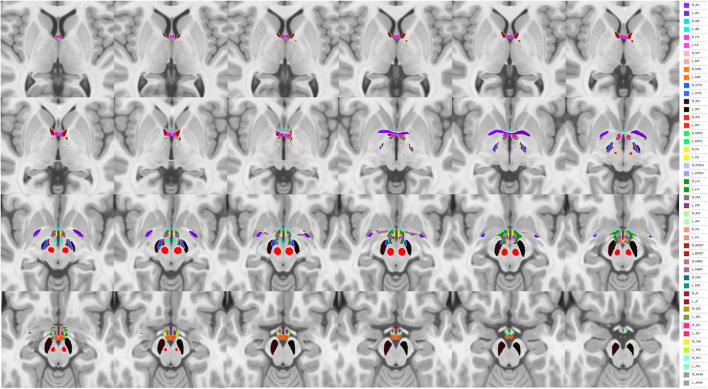
Projection of the Neudorfer hypothalamic atlas into a homemade 7T MP2RAGE Uni-Den template. Ac, anterior commissure; AHA, anterior hypothalamic area; AN, arcuate nucleus; BNST, bed nucleus of stria terminalis; dB, diagonal band of Broca; DM, dorsomedial hypothalamic nucleus; DPEH, dorsal periventricular hypothalamus; FX, fornix; LH, lateral hypothalamus; MM, mammillary bodies; MPO, medial preoptic nucleus of the hypothalamus; MT, mammillothalamic tract; NBM, nucleus basalis of Meynert; Pa, paraventricular nucleus; Pe, periventricular nucleus; PH, posterior hypothalamus; RN, red nucleus; SC, suprachiasmatic nucleus; SN, substantia nigra; SO, supraoptic nucleus; STN, subthalamic nucleus, TM, tuberomammillary nucleus; VM, ventromedial nucleus; ZI, zona incerta.

In this context, spatially more resolved raw data, brought by *in vivo* ultra-high field (UHF) 7T MRI, should play a major role in the finer characterization of HT nuclei at the individual level. Indeed, it should open new opportunities to better understand individual structural organization of tiny HT sub-structures. Thus, unbiased MP2RAGE acquisitions performed at 7T (55) should provide T_1_ weighted images and unbiased quantitative T_1_ maps at high spatial resolution (typically 0.6 x 0.6 x 0.6 mm) and allow for a better description of HT sub-structures. As an example, projection of the Neudorfer hypothalamic atlas to a home-made 7T MP2RAGE Uni-Den template on a single subject is presented in Figure 3.

**Figure 4:**
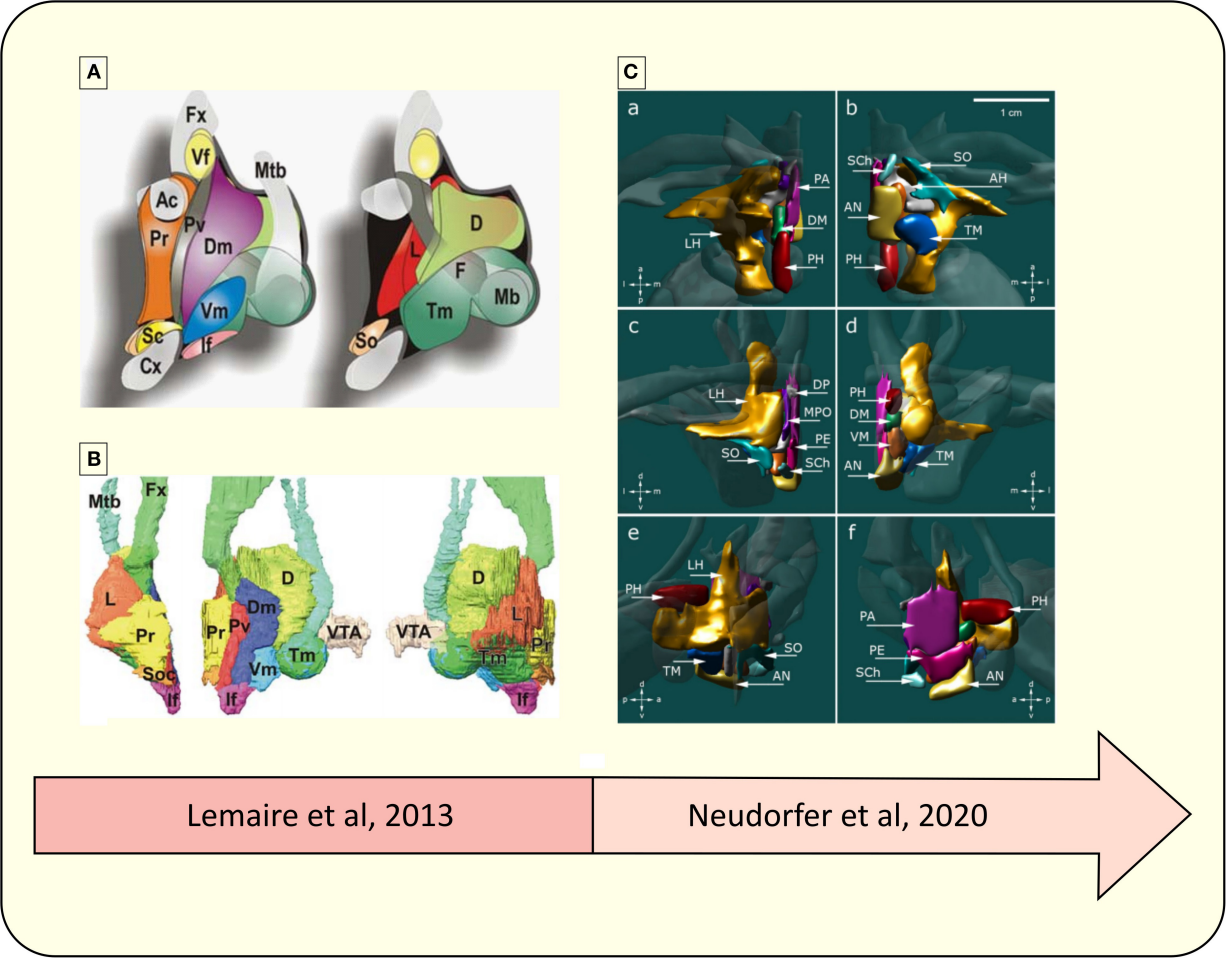
Representative diagram of the advances in the parcellation of the hypothalamus. **(A)** Schematic drawing of hypothalamic nuclei, lateral view from the midline. Left, nuclei directly located under the wall of the third ventricle; right, more deeply located nuclei up to the lateral region (52). **(B)** 3D overview of hypothalamic nuclei, constructed from a 3D high-field MRI data set. Left, frontal view; middle, lateral view; right, medial view. VTA, ventral tegmental area; Ac, anterior commissure; D, dorsal or posterior nucleus: Dm, dorsomedial nucleus; Fx, fornix; F, fornix nuclei; If, infundibular (arcuate) nucleus; L, lateral nucleus; Mb, mammillary body; Mtb, Fmillo-thalamic bundle; Cx, optic chiasma; Pv, paraventricular nucleus; Pr, preoptic nuclei; Sc, suprachiasmatic (ovoid) nucleus; Vf, ventricular foramen; So, supraoptic (tangential) nucleus; Tm, tuberomamillaris (mamilloinfundibularis) nucleus; Vm, ventromedial (tuber principal) nucleus (52). **(C)** 3D reconstruction of hypothalamic nuclei and their neuroanatomical relationships. (a) top view, (b) bottom view, (c) frontal view, (d) occipital view, (e) sagittal view depicting the outer surface of hypothalamic nuclei, (f) sagittal view depicting the inner surface of hypothalamic nuclei. AH, anterior hypothalamic area; AN, arcuate nucleus; DP, dorsal periventricular nucleus; DM, dorsomedial hypothalamic nucleus; LH, lateral hypothalamus; MPO, medial preoptic nucleus; PA, paraventricular nucleus; PE, periventricular nucleus; PH, posterior hypothalamus; SCh, suprachiasmatic nucleus; SO, supraoptic nucleus; TM, tuberomammillary nucleus; VM, ventromedial nucleus (4).

Figure 4 compares two different parcellation processes of the human HT using MRI.

In line with these technological improvements regarding acquisition and post-processing methods, the finer structural characterization of HT nuclei using UHF MRI should play a major role in the depiction of structural abnormalities in the different HT nuclei of patients with eatingdisorders.

## Anatomical Characterization oF HT in Obesity and Anorexia Nervosa

Very few structural hypothalamic abnormalities have been observed in obesity, while whole brain morphometry showed subtle gray matter (GM) atrophy in some regions of the frontal lobe implicated in behavioral control such as the post-central gyrus, the frontal operculum as well as the putamen which is involved in the regulation of taste and reward (56–58). BMI has been positively associated with GM volume in the right middle occipital gyrus involved in visual processing and systematically activated in food-cue task (57). Such high GM volumes of occipital regions of obese individuals could be associated to the selective attention bias toward appetitive food cue (59) and could constitute a predictor of future weight gain (57). In contrast, total body fat appeared negatively correlated with volumes of subcortical GM structures, with greater associations in men compared to women (58).

On the other hand, a lot of structural hypothalamic abnormalities were found in AN. HT GM volume has been found to be correlated with BMI in AN patients (60). Moreover, while a higher decrease in GM volume was observed in the cerebellum of AN patients with longer disease duration (> 9 years), lower hypothalamic GM was found significant only in AN patients with shorter disease duration (<3 years) (60).

Furthermore, a meta-analysis of voxel-based morphometry in AN revealed regional GM decreases in the left HT and other areas implicated in somatosensory perception and appetite, such as the left inferior parietal lobe, right lentiform nucleus and right caudate (61). Nevertheless, and surely due to the lack of spatial resolution, the largest variations were observed outside the HT, with enlargement of ventricles, cortical sulci (62), interhemispheric fissure and generalized volume reduction (63) associated with cortical thinning (64). GM atrophy has been specifically observed in somatosensory and reward regions which could explain abnormal reward responses to food stimuli and distortion of body perception (61). However, the nutritional status in AN affects cortical folding (65), and a longitudinal study on adolescent and young adults with acute and recovered AN patients reported that weight restoration seems to have a rapid reversible effect on cortical thinning (66).

## Structural Connectivity of Hypothalamus

The appetitive and self-control brain networks underlying food intake regulation can be represented by three interconnected sub-networks. First, the “lipostat” regulating energy balance signals including the HT, the ventral tegmental area (VTA) and the Substantia Nigra (SN) participating in the dopaminergic system. Second, the “limbic system” receiving sensory inputs and programming action to fulfill energy. This sub-network is composed by the amygdalar and hippocampal formations (memory, emotion, learning), the insula (ingestive cortex), the OFC and the ventromedial prefrontal cortex (VMPFC) (eating value), the accumbens nuclei and the striatum (motivation and value to action). Finally, the “control system” is composed by the dorsolateral prefrontal cortex (DLPFC) and the anterior cingulate cortex (ACC) which participates in self-regulation (67). Through the participation of its numerous nuclei and their WM connections, the HT appears as a central node for each of these different sub-networks.

Diffusion-weighted MRI has helped to better characterize *in vivo* this organization. Kamali and coworkers showed that the stria terminalis connects the amygdala to the anterior HT (68). The stria terminalis projects to the mammillary body region as well as multiple septal and hypothalamic nuclei, which could not be identified due to the lack of spatial resolution (68). Atlases of human neuroanatomy reported that the ventral amygdalo-fugal fibers originating from the basolateral and central nucleus of the amygdala, project to the nucleus accumbens, basal forebrain, medial dorsal nucleus of the thalamus (69). The ventral amygdalo-fugal fibers also reach the septal nuclei, LHA, lateral preoptic, anterior and tuberal nuclei of the HT (70, 71).

The fornix originates from the hippocampal formation and links the hippocampus to major structures involved in the homeostatic (HT), hedonic valence (amygdala) and reward control (OFC, nucleus accumbens) of food intake (12, 68, 72). The fornix fibers were also shown to terminate in the region of mammillary bodies and septal nuclei (68). The pathway connecting the hippocampus to the HT might be involved in the motivated and conditioned pattern of food intake (73).

Recently, Kamali's team was the first to reconstruct the dorsal thalamo-hypothalamic tract, revealing in the meantime the existence of a direct connection from the dorsal thalamus to the ventral hypothalamic nuclei (74). The authors hypothesized that since the thalamus constitutes a hub for somatosensory information, the dorsal thalamic nuclei could carry sensory information from the thalamus to the limbic system, in particular to the HT. These findings are in line with studies done in rats suggesting the association of the thalamus and the HT with eating behavior, food motivation/ reward and the circadian rhythms (75–77).

A high-spatial and angular resolution diffusion weighted tractography technique revealed the detailed pathway of the parieto-occipito-hypothalamic tract. The parieto-occipito-hypothalamic tract seems to allow for visuosensory information to be carried from the parietal and occipital cortices to the limbic system, especially the HT and the bed nucleus of the stria terminalis. This finding stresses the point that many limbic functions, such as hunger, fear or circadian rhythm rely on visuo-sensory information (78).

The cerebellum might be involved in the locomotor and foraging aspects of eating behavior (79). Lesional animal studies showed that ablation of the cerebellar nuclei induced degeneration in the HT, reflecting the existence of a cerebello-hypothalamic connection (80, 81). In human, high-resolution diffusion-weighted tractography at 3T revealed that the cerebellar-ponto-hypothalamic tract is responsible for the reciprocal communication between the cerebellum and the limbic system, especially the HT and the septum (82). More recently, a direct cerebello-hypothalamic tract was described in Human. Indeed, DTI results reported fibers projecting from the cerebellar nuclei to the contralateral anterior HT and ipsilateral posterior HT (83). The cerebellar-hypothalamic connection seems to allow for the convergence of non-limbic information to the limbic system through the hypothalamic and septal nuclei. Numerous animal studies support the modulatory action of the cerebellum on food intake through its action on the HT (84). Therefore, these findings suggest that the cerebellar-ponto-hypothalamic tract might carry the modulatory action of the cerebellum on hypothalamic feeding centers.

**Figure 5:**
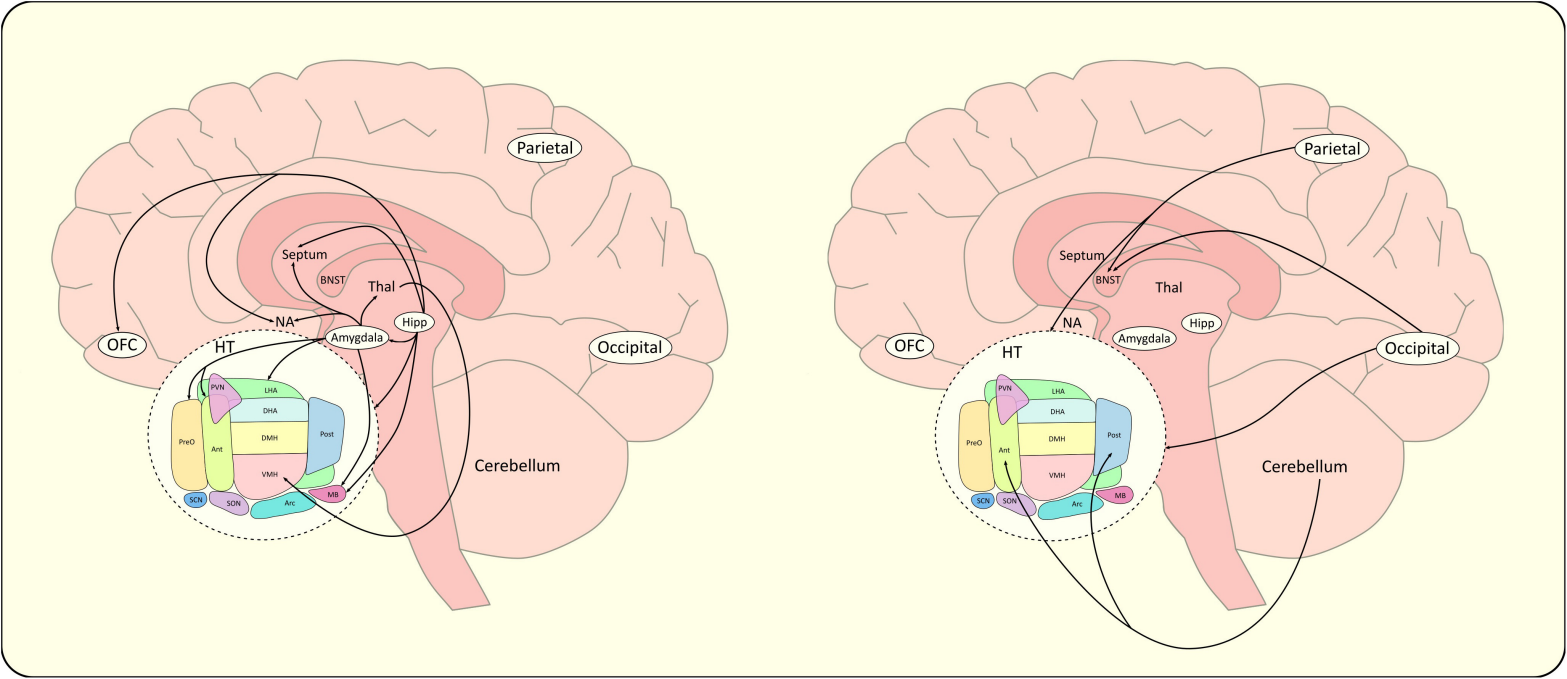
Schematic representations of extra-hypothalamic networks of food intake. For clarity concern, extra-hypothalamic connections reported in the literature have been divided in an appetitive network **(left)** and a control network **(right)**. BNST, bed nucleus of the stria terminalis; Hipp, hippocampus; HT, hypothalamus; NA, nucleus accumbens; Parietal, parietal cortex; Occipital, occipital cortex; OFC, orbitofrontal cortex; Thal, thalamus. The schematic sagittal slice of the hypothalamus (HT) includes; Ant, anterior hypothalamus; Arc, arcuate nucleus; DHA, dorsal hypothalamic area; DMH, dorsomedial hypothalamus; LHA, lateral hypothalamic area; MB, mammillary body; Pe, periventricular nucleus; Post, posterior hypothalamic nucleus; PreO, preoptic nucleus; PVN, paraventricular nucleus; SCN, suprachiasmatic nucleus; SON, supraoptic nucleus; VMH, ventromedial hypothalamus.

Finally, structural connectivity derived from DTI tractography was used to parcel the HT by studying the orientations and projections of the different efferent and afferent WM fibers connecting the HT to other brain areas. Lemaire and co-workers characterized parts of the macroscopic internal structure of the HT and its extrinsic connectivity with deep brain and cortical regions (85). Thus, preoptic, anteroventral and lateral hypothalamic regions were shown to predominantly project to the right frontal hemisphere, largely involved in behavioral control (85). Figure 5 summarizes the structural extra-hypothalamic networks of food intake discussed above. Using color coded directional fractional anisotropy (FA) maps of the HT, three different subregions of the HT were defined: an anteromedial region with dorsoventral diffusion direction, a posteromedial region with rostro-caudal direction, and a lateral region with mediolateral direction (86). The anteromedial region was assigned to the PVN, anterior, and dorsomedial hypothalamic nucleus and part of the LHA, the lateral region to the VMH and supraoptic nucleus and finally the posteromedial region to the suprachiasmatic, infundibular, VMH, posterior hypothalamic, medial and lateral mammillary nucleus.

## Altered Structural Connectivity in Obesity and Anorexia Nervosa

Obesity has been associated with abnormal WM volume and microstructure as well as microstructural brain diffusion changes in HT and other structures involved in satiety and hunger (87). Indeed, DWI studies revealed that the apparent diffusion coefficients (ADCs) of HT, hippocampal gyrus, amygdala, insula, cerebellum and midbrain were significantly increased in obese patients. More specifically, hypothalamic longitudinal diffusivity λ_1_, a DTI metric related to axonal integrity, has been found to be negatively correlated with fat mass in obese subjects (88). Furthermore, altered hypothalamic microstructure, characterized by a higher mean diffusivity (MD) within the HT, has been associated with a higher BMI (89). Indeed, alteration in the hypothalamic microstructure visible on DTI has been associated with the presence of inflammatory factors, such as C-reactive protein (CRP) or lipopolysaccharide-binding protein (LBP) (88). However, there is no consensus regarding the sign of the correlations between BMI and WM volumes. Most studies have established an increase of WM volume in the frontal, temporal, parietal and occipital lobes, fusiform and parahippocampal gyri, brainstem and cerebellum in obese relative to lean participants (90, 91). On the contrary, a decrease in WM volume has been reported in overweight and obese older adults within the frontal lobe, anterior cingulum and corona radiata (92). The percentage of total body fat has been positively associated with global FA in men and women, and negatively associated with global MD in women. These results suggest that obese women globally present higher integrity but lower magnitude of WM microstructure, showing differential gender effects of obesity on WM microstructure (58). Generally, the association between markers of obesity and diffusion parameters seems stronger in women than in men (93). However, regionally, obese individuals demonstrate a negative correlation between BMI and FA in the corpus callosum and fornix (94). Furthermore, BMI was found to be negatively associated with the microstructural integrity of numerous WM bundles such as callosal and limbic tracts (95). Interestingly, obese subjects show partial recovery of WM volumes after only 6 weeks of dieting (90).

Alteration of WM microstructure has also been observed in AN patients (96). DTI studies reported low FA and/or high MD of the fornix, amygdala and the prefrontal region in AN (97). Low FA was also found in the lateral part of the cerebellum of AN patients (84, 98). This result is in line with animal studies stating that cerebellar nuclei would modulate lateral hypothalamic response to satiety and hunger through interaction with gastric vagal nerves (99).

Using a probabilistic tracking algorithm, the same team who implemented the first 3D-MRI atlas of the HT (50) found a significant reduction in the number of fibers in the arcuate nucleus of AN patients and leanness controls compared to normal-weight controls but higher connectivity of LHA in AN compared to leanness and normal-weight controls (42). In the same study, high basal levels of HT glutamatergic markers (Glutamate-Glutamine/Cr ratios) derived from magnetic resonance spectroscopy (MRS) dropped abnormally following food intake in AN compared to controls (42). Therefore, lower BMI seems to be associated to a specific altered structural connectivity of the arcuate nucleus and LHA as well as a different HT metabolic response to food intake (42).

Moreover, AN seems to be characterized by a disrupted fronto-accumbal structural connectivity since a longitudinal neuroimaging study revealed that the abnormal increase in structural connectivity within the fronto-accumbal network found in AN patients persisted even after weight recovery (100).

However, the full structural connectome of HT nuclei is far from being entirely described, both at the intra-HT and extra-HT connectivity levels. DTI techniques cannot provide crucial information such as the afferent or efferent nature of fiber tracts. Only significant improvements in DWI acquisitions and models including high angle resolution diffusion imaging (HARDI), orientation diffusion functions, multi-shell acquisitions and/or UHF protocols increasing spatial resolution (101), and correlation with post-mortem data should allow for the full characterization of these complex hypothalamic networks. In that sense, improvement of post-processing pipelines validated in large cohorts (102) will help to correct distortion artifacts (eddy currents, susceptibility effects, motions…) in UHF DWI and will allow for high quality data to be accurately overlaid with HT nucleus structural atlases.

## Functional Connectivity of Hypothalamus

Appetite networks have also been explored through the prism of functional connectivity (FC) by using resting-state functional MRI (rs-fMRI). rs-fMRI consists in recording temporal correlations of low frequencies blood oxygen level dependent (BOLD) fluctuations between various brain areas during a “resting” period (103). This technique is commonly used to identify functional networks when no task is performed (104).

Such paradigm was used to explore the effect of fasting and satiation on the FC patterns in healthy subjects (105). Interestingly, variations of blood glucose levels between scans accounted for numerous altered functional connectivities. Indeed, FC between the left HT and inferior frontal gyrus strengthened during fasting whereas FC increased between the right HT and the superior parietal cortex during satiation (105).

In healthy subjects, a depleted energy state seems to favor connectivity of the HT with the inferior frontal gyrus, which is implicated in suppressing the desire for food and preventing temptation to eat (106, 107). On the contrary, satiation is associated with a stronger connectivity between the HT and the superior parietal cortex, which is hypothesized to be involved in decreasing the eating drive by being less distracted by thoughts of food or eating (105). Thus, the HT connectivity seems to be lateralized and to depend on the energy state.

Hunger may increase effective connectivity (EC) from posterior insula to anterior insula while EC from the anterior insula to posterior insula may be decreased (108). Therefore, individuals' energy states affect the strength and directionality of connections between sub-regions of the insula. However, this same study failed to provide evidence of an effect of hunger on the connection between the HT and the insula.

Seed-based resting-state connectivity maps derived from a 3T MRI database of 49 healthy subjects were generated from the LHA (109) considered as the hunger center, and from the medial HT (including the VMH) considered as the satiety center (11, 110). The LHA was shown to be strongly connected to the dorsal striatum (caudate, putamen), anterior cingulum, thalamus, and frontal operculum, regions involved in goal-directed behavior (111), and to the lateral OFC implicated in coding stimulus reward value (112). The LHA was also shown to receive preferentially inputs from the lateral OFC (113). In contrast, the medial HT showed stronger connections with the ventral striatum (nucleus accumbens), the medial OFC and the occipital gyrus, regions involved in reward and motivation (114) and with the medial OFC (113).

Improvement of statistical power through a better temporal resolution and higher spatial resolution available with UHF MRI scanners (115) should be a game changer in the study of functional reorganization of HT networks in healthy subjects and eating disorders patients.

## Modulation of FC in Obesity and Anorexia Nervosa

Functional connectivity is altered in obese patients when compared to healthy subjects. On one hand, high BMI subjects show a decreased global brain connectivity (GBC) in the lateral and prefrontal cortices, the insula and the limbic system (116), and a reduced FC between HT and regions involved in cognitive control such as the superior parietal lobule (117). On the other hand, obese patients show increased global brain connectivity in the visual, parietal, and premotor cortices (116). Functional connectivity of the LHA with the ventral striatum, anterior insula and OFC were found to be enhanced in obese compared to healthy individuals (118). In addition, high BMI subjects show increased FC between HT and regions involved in motivation processes (insula, thalamus, globus pallidus, cerebellum) (117), and increased FC between medial HT and regions involved in reward processing such as OFC and nucleus accumbens (109). Therefore, BMI is positively associated with FC between HT and brain regions involved in motivated feeding, reward processes and motricity, while FC between HT and areas involved in cognitive control of food intake is negatively correlated to BMI (117).

Modulation of hunger states showed that obese patients exhibit higher connectivity between the HT and the medial prefrontal cortex (PFC) and the dorsal striatum when fasted compared to lean subjects (119). Furthermore, in contrast to lean subjects, FC between HT and the PFC did not decrease when fed, which could explain the increased craving for food obese individuals experience during a fast (119). Additionally, lower FC was found between the HT and brainstem in response to glucose administration in obese compared to normal-weight control (120). These results stress the presence of FC alterations between the HT and regions involved in the inhibition of the eating drive, thus limiting the control of the balance between energy intake and expenditure (119). However, weight loss surgery allowed reversibility of FC abnormalities within HT networks, with restored lower FC between the HT and the OFC and somatosensory cortices (121).

Even though numerous resting-state fMRI studies were conducted in AN, most of them did not look at HT connectivity. Indeed, they rather focused on altered local- and large-scale- connectivity of “conventional” resting state networks (RSN) such as the default mode network (DMN), the visual network and auditory networks, as well as the reward and cognitive control systems. The DMN is a network associated with stimulus-independent thoughts and self-reflection (122). Within this network, increased FC was observed between the dorsal ACC and regions such as the precuneus and the retro-splenial cortex, which was positively correlated with body shape questionnaire scores (123). Such FC increase could represent a supplementary effort of the cognitive control system to restrain appetite (124). Data driven approach using independent component analysis (ICA) showed increased FC between the angular gyrus and the frontoparietal network in AN, which was also correlated with stronger cognitive control scores (125). In AN patients compared to healthy subjects, EC was increased from the bilateral orbitofrontal gyrus to the right IFG and from the bilateral insula to the left IFG while EC was decreased from the right inferior frontal gyrus to the mid-cingulum (109). These findings suggest that connectivity within the control network is reduced whereas connectivity between salience-related systems is increased in AN.

The ventral attentional circuit was also shown to be abnormally highly connected in AN, especially the inferior frontal gyrus (126). Since the ventral attention circuit allows for the implementation of appropriate behavioral responses, especially when an unexpected or salient stimulus occurs, its alteration could be responsible for lower interoceptive sensations awareness and struggles in adapting one's behavior to environmental needs (127). Visuospatial and auditory processing involved in self-body image have also appeared to be disturbed in AN patients, with reduced FC within the lateral visual cortices and the auditory network (104), and between the sensorimotor and visual circuits (128).

Furthermore, hyperconnectivity in AN of the dorsal caudate with prefrontal regions involved in motor and cognitive functions (129), have been interpreted as altered habit learning deficiencies responsible for the maintenance of AN symptoms (130).

Within the reward system, AN patients showed increased FC between the left nucleus accumbens and the left medial OFC compared to control subjects (100). Moreover, the connectivity within the ventral fronto-striatal circuit also seems to be dysfunctional in AN. Furthermore, hypoconnectivity between nucleus accumbens and superior frontal gyrus, found in AN, correlated with greater cognitive eating disorder symptoms (Eating Disorder Examination Global scores). Collectively, these findings suggest that FC is altered in AN in key brain regions involved in reward processing, homeostatic communication and habit.

Half of the AN individuals who received medical care need to be re-hospitalized within a year of discharge (131). Given the persistence and high risk of relapse of this long-term disease, the study of the resting state functional connectivity (rsFC) in weight-recovered AN individuals is particularly important.

Complete restoration of rsFC in weight-recovered AN individuals for at least 12 months has been observed in the PFC, sensorimotor, precuneal, insular, left parietal and left temporal cortices (132). In contrast, altered FC persists after 6 months recovery in the frontoparietal network with reduced FC with the DLPFC and increased FC with the angular gyrus and between the precuneus and DLPFC (133). Overall, rsFC alterations in AN tend to normalize after weight restoration, with the noticeable exception of the cognitive control network, which could in part explain the high risk of relapse of this disease.

However, more resting-state studies are needed since findings on the same networks are inconsistent (134). Moreover, undernutrition was found to alter reward and habit learning related networks (135) as well as to influence rsFC results (108). Therefore, rsFC AN studies might observe the consequence of starvation and not the neuronal correlates of AN (136). On the other hand, it can be hypothesized that a malfunction of the reward system could be the trigger of AN in genetically predisposed individuals exposed to certain environmental conditions such as emotional shock or diet [see (137) for review]. Furthermore, most studies did not take into account the different subtypes of AN (restrictive and binge eating/purging), although they were not found to influence results (100).

## Task Related fMRI of Eating Disorders and Hypothalamus

Different methods of functional imaging have been used to study appetite networks. Tracking variations of BOLD signal (fMRI), regional cerebral blood flow (rCBF) (PET or fMRI) or ADCs (diffusion weighted MRI) have been conducted (i) during different hunger states, (ii) before, during and after glucose (or other nutriment) ingestion, or (iii) during different food cue image display paradigms, including (or not) different satiety states.

Hunger was shown to enhance BOLD signal and rCBF within the HT, the ACC, the insula, the parahippocampal gyrus, and the hippocampus, core regions of the limbic system (138). Concurrently, during satiation, increase in rCBF was observed in the VMPFC, the DLPFC, and the inferior parietal lobe, which are all involved in the control of food intake (138).

Glucose ingestion in healthy subjects induce a dose-dependent decrease in the BOLD signal within the PVN and the VMH, which have an anorexigenic role (139). Besides, oral glucose intake triggers two peaks of response in healthy subjects: one just after the ingestion, followed by another peak approximately 10 min later (140). This delayed response was found to be associated with a negative response in the medial HT, and the fasting plasma insulin concentrations (140) reflecting the important role of HT in the modulation of insulin secretion to regulate glucose levels (141).

The type of ingestion may also play a role since both sweet taste and energy content are necessary to trigger a hypothalamic response (142). However, the limited spatial resolution of these techniques may prevent the ability to detect such changes in HT during glucose ingestion due to the different roles and activity patterns (stimulatory or inhibitory) of close-by hypothalamic nuclei (143).

However, more recently, Osada and colleagues described the glucose metabolism of individual major HT nuclei by using areal parcellation based on areal profiles of resting state functional connectivity (144). They observed a decreased activity in the VMH but an increased activity in the LHA between 10 and 40 min after glucose ingestion. Furthermore, the decrease in activity found in the arcuate nucleus was followed by a rise in blood insulin during the first 10 min after glucose ingestion (144).

Sun and colleagues demonstrated that the amygdala response to the administration of a milkshake predicted weight in satiated but not in hungry individuals (145). Looking at EC, sated subjects showed an unidirectional gustatory input from basolateral amygdala to the HT, whereas during hunger, HT drove bilateral connectivity with the amygdala (145).

Another popular neuroimaging method commonly used to investigate appetite networks is food-cue reactivity paradigms, which consist in presenting images of food under various forms and energy-content as well as non-related food images. Comparison of BOLD signals between conditions helped to decipher appetite processes. Indeed, high-calorie food were shown to trigger a significant bilateral activation of the HT (146, 147), the medial and DLPFC, the medial dorsal thalamus, the cingular cortex, and the cerebellum compared to the non-food condition (146). The cerebellum was also shown to be activated when viewing high fat relative to low fat food images (146). Furthermore, numerous animal studies revealed that the cerebellum modulates the activity of the LHA through its connections with gastric vagal nerves involved in hunger and satiety signaling (99).

Similarly to results obtained during the glucose ingestion, presentation of rewarding food tastes and images during satiation decreased BOLD signal in the reward system, including the HT, vmPFC, nucleus accumbens, OFC, and insula but increased BOLD response in the DLPFC, which is part of the control network (89). Similar manipulation of food types through hunger states showed that presentation of high-energy food during fasting might activate the HT and ventral striatum to a greater extent compared to low-energy food (148). fMRI is also used in the study of emotional connotation of food, which could be a factor in unhealthy food choices. One of this studies found an association between lower food healthiness evaluation of food in a food-cue task, and higher activation in the amygdala (149). Therefore, the ability to differentiate healthy from unhealthy foods is affected by emotional state.

Nevertheless, reproducibility across these studies appears to be highly dependent on various external factors, limiting the fine characterization of the role of HT within food intake networks. One meta-analysis (148) revealed that no more than 41% of experiments contributed to the activated voxel clusters for the contrast between food and non-food pictures, which could be explained by biases of subject selection according to age (150), BMI scores (151), sex, hunger states (152), emotional state (153), dietary restriction (154) and subject genotype (155). Minimization of potential biases relative to menstrual cycle, which could trigger cravings, has been proposed through the recommendation of performing MRI scans during the follicular phase (156). Indeed, mean diffusivity and metabolism of HT appear to be modulated by artificial menstrual cycle, leading to a decrease in ADCs and increase in Choline/NAA ratios after oral contraceptive use compared to pill free period in young healthy women (157).

Regarding study designs, food paradigms differ between studies in the nature and nutritional properties of the food presented, in task instruction and even in sample sizes, which are generally small, and differences in the acquisition and analyses methods (148). In regard to these irregularities, which hinder the understanding of the role of the HT in the regulation of food intake, standardized databases of food and non-food images have been implemented (158, 159). Moreover, the duration of the fasting period before scan sessions should be harmonized to create a more homogenous hunger state in subjects across studies.

Recent studies have tried to minimize the bias related to individuals' food preferences. Some studies take into account the participant's preferred food in their script (160), while others ask them to rate the appetence of the presented food (161). One study even had volunteers rate their hunger, satiety, thirst, fullness and emptiness, before and after the scanning session (162). However, taking into account those ratings did not refine the model fit in this food-cue reactivity study.

## Functional Imaging In Obesity

During satiation, obese individuals showed deactivation of the precuneus and the superior parietal cortex, with lower rCBF in the HT, cingulate, nucleus accumbens, in the limbic and paralimbic regions (parahippocampal gyrus, insular cortex, amygdala), caudate nucleus, frontal and temporal regions relative to controls (25, 163). In contrast, sated obese women exhibited significantly higher rCBF in the ventral PFC and frontal operculum (25, 163). These findings are in agreement with the hypothesis that the PFC is implicated in the termination of a meal by its inhibiting action on limbic and paralimbic areas (25).

Concurrently, glucose ingestion in obese patients relative to controls induced a delayed and attenuated dose-dependent decrease in BOLD signal in the PVN and VMH (139). In addition, the decrease of rCBF in HT during a meal associated with normal satiety processes in healthy subjects has appeared to be attenuated in obese patients (163).

When administered a liquid meal, obese and former obese who succeeded in decreasing their BMI from at least 35 to a BMI of 25 kg/m^2^ and in keeping their weight stable for no less than 3 months before the start of the study, exhibited an increased rCBF in the middle insular cortex while no change in activity was registered in lean individuals (164). Additionally, when given a satiating amount of the same liquid, obese presented a different neural response in the posterior hippocampus, posterior cingulate cortex and amygdala. Indeed, the posterior hippocampus rCBF decreased in a similar manner in obese and former obese while it increased in lean individuals (164).

Persisting differential activities in regions implicated in gustation and rewarding/ hedonic aspect of food (insular cortex) or enteroception and learning/memory (hippocampus) could partly explain the high risk for relapse of former obese. Interestingly, a study on smokers and non-smokers revealed that the hypothalamic response to oral intake of milkshake in non-smokers was lower, which is associated with long-term weight change in this population (165).

Food-cue task fMRI studies have reported elevated responsivity of somatosensory (Rolandic operculum), gustatory (insula, frontal operculum) and reward brain areas (caudate, putamen, amygdala, OFC) in obese subjects compared to lean individuals when presented with palatable food cues (166–168). Abnormal activity patterns in obese patients have been found in the HT, PFC, parietal and temporal cortex, cingulate cortex, nucleus acumens, amygdala, midbrain, insula, and OFC (25, 164).

During high-calorie food cue, obesity has been associated with hyperactivation of the right putamen, left caudate body, left anterior insula, left hippocampus, and the left parietal lobe relative to control, while low-calorie food cue elicited exacerbate responses in the left superior frontal, right middle and inferior frontal gyrus, middle occipital gyrus, and left superior temporal gyrus in obese compared to healthy controls (166). Thus, BMI was positively associated with higher response to high-calorie in brain regions involved in the processing of taste information (anterior insula, lateral OFC), motivation (OFC), emotions and memory (posterior cingulate cortex), salience-guided orienting (claustrum) and reward anticipation (dorsal striatum) (166, 169). BMI is also positively associated with selective attention toward appetitive food-cue and higher responsivity in reward regions such as the ventrolateral prefrontal cortex (VLPFC), lateral OFC, temporal operculum and anterior insula (59, 170). However, when asked to inhibit responses to high-calorie food images, obese individuals exhibited less activation in frontal inhibitory regions, such as the superior and middle frontal gyrus, VLPFC and VMPFC, and OFC, relative to lean individuals, which is consistent with behavioral evidence of impulsivity often found in overweight individuals (170). These findings suggest that a higher weight is preferably linked to hypo-activation of inhibition control regions and hyper-activation of reward regions in response to palatable food. Failure of inhibition control regions to inhibit reward regions when presented with appetitive food could increase the susceptibility to overeat (171). Moreover, obese-like activity in the middle insular cortex and hippocampus persists in former obese (164). These results suggest that higher activation in reward and somatosensory regions in response to food-cue constitute a risk for future weight gain.

During reduced weight maintenance, obese individuals show high FC between the HT and visual (occipital fusiform and temporal fusiform areas, superior lateral occipital cortex and cuneus), memory (hippocampus) and attention areas (dorsal ACC, left middle and inferior frontal gyri) when viewing food compared to non-food images (172). Moreover, the inferior frontal gyrus, implicated in inhibitory control (173), has been found to be more activated by food images in people able to maintain their weight loss than in obese or lean subjects (174) and its activation in satiation is lower in obese than in normal weight participants (175). These results suggest that during weight loss, obese patients have a higher sensitivity to food cues. Furthermore, the inhibitory action of the inferior frontal gyrus seems to be necessary to maintain weight loss (174).

Overall, obesity seems to be associated with a concurrent blunted hypothalamic reactivity to glucose ingestion and an exacerbated hedonic reactivity, which do not coincide with homeostatic state and lead to overconsumption of high fat food.

## Functional Imaging In Anorexia Nervosa

Considering the ability of AN patients to fight against the natural instinct to feed oneself, the hypothesis of a dysregulation in hypothalamic glucose-sensitivity was investigated. AN and controls exhibited the same decrease in neuronal activity when administered with a glucose solution (176). Therefore, the hypothalamic reactivity to glucose does not seem to be altered in AN. However, contrary to normal-weight controls and obese subjects, AN patients did not display an increase of FC between the HT and brains regions implicated in reward processes (amygdala, nucleus accumbens) during infusion of water when compared to glucose infusion, with the exception of the posterior insula (120). As opposed to control, AN did not show deactivation in the mesocorticolimbic reward circuit including structures such as the caudate nucleus, putamen, insular cortex, medial OFC, and inferior operculum after glucose administration (120).

Furthermore, AN exhibited a higher FC between the HT and the left ventral striatum in response to glucose administration relative to normal-weight controls and obese subjects (120). The abnormal hyper-activation of the ventral striatum is a neural signature of patients suffering from AN and is hypothesized to be involved in the onset and maintenance of this illness (177). Interestingly, a study comparing the response to pictures of underweight women demonstrated a higher activation in the left ventral striatum in AN patients compared to lean control (178).

Moreover, patients with AN do not show satiety state–dependent connectivity between the HT and the meso-corticolimbic reward circuit that is commonly found in normal-weight controls. This observation could explain the reduced hedonic coding aspect of food associated with AN (120). AN individuals show hypoactivation of the food motivation network in response to high-calorie foods (179). Indeed, both fasted women with weight-restored (maintenance of 90%−110% ideal body weight for at least 6 months) and active AN exhibited hypoactivity in the HT, amygdala and anterior insula in response to high-calorie foods vs. objects when compared to control. Hypoactivation in the anterior insula persisted in women with active disease after they were served a meal (18% calories from protein, 23% from fat and 59% from carbohydrates).

In contrast, weight-restored AN women show similar activation to controls after a meal (179). Compared with controls, women with active disease show hypoactivation in the HT, amygdala, hippocampus, OFC and anterior insula when fasted and in the amygdala and insula after they ingested a meal (179). Interestingly, activations in the HT, amygdala and anterior insula are significantly associated with appetite (hunger) and appetence ratings (how appealing is the food) in controls and weight-restored AN women but not in those with active disease (179). The authors provided evidence of a dysfunction of the food motivation network, including the HT, in women with active AN, which tends to persist even after weight-restoration. AN has been shown to be associated with lower food-cue processing activity in brain structures implicated in reward and salience (HT, striatum, hippocampus, amygdala, cerebellum, insula) but greater activity in regions involved with cognitive control (dorsolateral PFC, mPFC, OFC, ACC) (180, 181). To sum up, AN has been associated with a reduced hypothalamic reactivity and connectivity with the reward system, which could be responsible for the repressed motivational responses to food cue, in relation with an alteration in food reward processing.

## Conclusion

Despite the important progress in the neuroimaging of HT and the better characterization of its functional activation pattern across different hunger states or eating disorders, its role within the structural and functional food-intake networks remains to be elucidated. UHF MRI combined with the use of the highly resolved atlases (4) should allow for a better understanding of the role of each hypothalamic nuclei in a normal and pathological context. Indeed, advanced magnetic resonance (MR) techniques provide new significant anatomical, functional metabolic and connectomic insights of the HT, through the improvement of quantitative MRI, spectral/spatial resolution of MRS, spatial resolution of anatomic MRI, diffusion and functional MRI. Furthermore, the current paper briefly mentioned the possible contribution of metabolic MRI in the study of obesity. In addition, MRI is an undeniable interesting tool in the study of the effectiveness of treatments for eating disorders in humans since this technic is non-invasive and partially replace for the lack of AN animal models. Finally, combining the study of the HT at the structural, functional and metabolic levels could bring new perspectives to better understand and better treat patients with eating disorders, as it is already done in the study of pathologies such as Alzheimer's (182) or epilepsy (183).

## Author Contributions

All authors listed have made a substantial, direct, and intellectual contribution to the work and approved it for publication.

## Funding

CR had received a PhD grant from the Neuroschool, a support from the French government under the Programme Investissements d'Avenir, Initiative d'Excellence d'Aix-Marseille Université via A^*^Midex (AMX-19-IET-004) and ANR (ANR-17-EURE-0029) funding.

## Conflict of Interest

The authors declare that the research was conducted in the absence of any commercial or financial relationships that could be construed as a potential conflict of interest.

## Publisher's Note

All claims expressed in this article are solely those of the authors and do not necessarily represent those of their affiliated organizations, or those of the publisher, the editors and the reviewers. Any product that may be evaluated in this article, or claim that may be made by its manufacturer, is not guaranteed or endorsed by the publisher.
